# Turning Meiosis into Mitosis

**DOI:** 10.1371/journal.pbio.1000124

**Published:** 2009-06-09

**Authors:** Isabelle d'Erfurth, Sylvie Jolivet, Nicole Froger, Olivier Catrice, Maria Novatchkova, Raphaël Mercier

**Affiliations:** 1 INRA, UR254, IJPB, Versailles. France; 2 CNRS, UPR2355, Gif sur Yvette, France; 3 Research Institute of Molecular Pathology (IMP), Vienna, Austria; The University of North Carolina at Chapel Hill, United States of America

## Abstract

Apomixis, or asexual clonal reproduction through seeds, is of immense interest due to its potential application in agriculture. One key element of apomixis is apomeiosis, a deregulation of meiosis that results in a mitotic-like division. We isolated and characterised a novel gene that is directly involved in controlling entry into the second meiotic division. By combining a mutation in this gene with two others that affect key meiotic processes, we created a genotype called *MiMe* in which meiosis is totally replaced by mitosis. The obtained plants produce functional diploid gametes that are genetically identical to their mother. The creation of the *MiMe* genotype and apomeiosis phenotype is an important step towards understanding and engineering apomixis.

## Abstract 2

The mutation of as few as three genes in a sexual plant transforms meiosis into mitosis and results in diploid gametes that are genetically identical to the mother plant. This phenotype resembles apomeiosis, which is a major step in apomixis.

## Author Summary

In the life cycle of sexual organisms, a specialized cell division—meiosis—reduces the number of chromosomes from two sets (2n, diploid) to one set (n, haploid), while fertilization restores the original chromosome number. In contrast, mitosis produces two identical daughter cells. Basically, three features distinguish meiosis from mitosis. Meiosis has: (i) a succession of two rounds of division following a single replication, (ii) recombination, and (iii) co-segregation of sister chromatids at the first division. In this study, we identified a gene that controls one of these three features— entry into the second meiotic division—in the sexual plant *Arabidopsis thaliana*. By combining a mutation in this gene with two other mutations—one that eliminates recombination and another that modifies chromatid segregation—we created a genotype (called *MiMe* for mitosis instead of meiosis) in which meiosis is totally replaced by mitosis. As a consequence, *MiMe* plants produced diploid male and female gametes that are genetically identical to their parent, and ploidy doubles at each generation. The replacement of meiosis by mitosis is a key component of apomixis, or clonal reproduction through seeds, which has potential revolutionary application in crop improvement.

## Introduction

**Figure 1:**
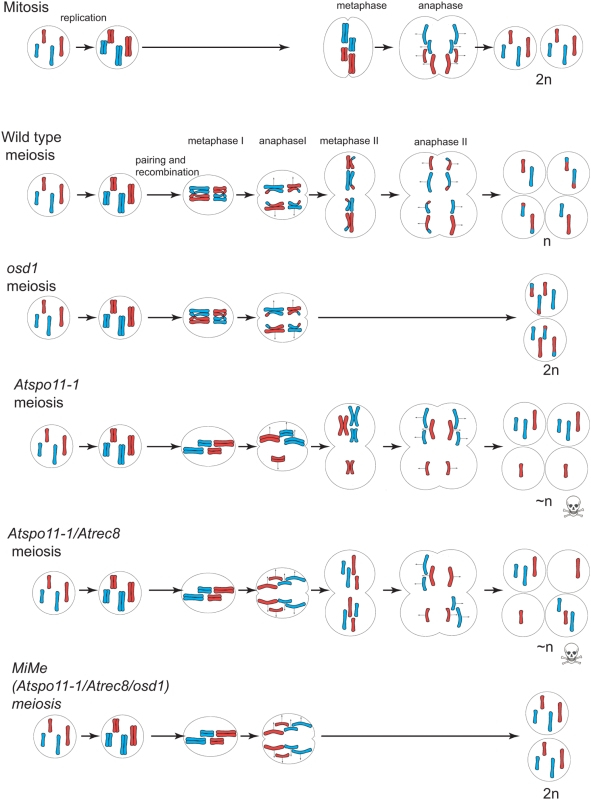
Schematic summary of the main results. During mitosis in diploid cells, chromosomes replicate and sister chromatids segregate to generate daughter cells that are diploid and genetically identical to the initial cell. During meiosis, two rounds of chromosome segregation follow a single round of replication. At division one, homologous chromosomes recombine and are separated. Meiosis II is more similar to mitosis, resulting in equal distribution of sister chromatids. The obtained spores are thus haploid and carry recombined genetic information. In the *osd1* mutant (this study), meiosis II is skipped giving rise to diploid spores and gametes with recombined genetic information. The double *Atspo11-1/Atrec8* mutant undergoes a mitotic-like division instead of a normal first meiotic division, followed by an unbalanced second division leading to unbalanced spores and sterility [9]. In the triple *osd1/Atspo11-1/Atrec8* mutant (*MiMe*, this study), the presence of the *Atspo11-1* and *Atrec8* mutations leads to a mitotic-like first meiotic division, and the presence of the *osd1* mutation prevents the second meiotic division from occurring. Thus meiosis is replaced by a mitotic-like division. The obtained spores and gametes are genetically identical to the initial cell.

Apomixis, or asexual reproduction through seeds, results in progeny that are genetic clones of the maternal parent [1],[2]. Apomixis is of great interest due to its potential application in crop improvement. By introducing apomixis into sexual plants, any desired genotype, no matter how complex, could be perpetuated through successive seed generations [3],[4]. However, despite the occurrence of apomixis in over 400 species of angiosperms, very few crop species are apomictic and attempts to introduce this trait by crossing have failed [4]–[6]. An alternative approach is to de novo engineer apomixis [3]. For this strategy to be applied, the genes that confer elements of apomixis must first be identified. One major element of apomixis is apomeiosis, the skipping or deregulation of meiosis resulting in a mitotic-like division, which prevents ploidy reduction, and means that all the parent's genetic information is retained in the gamete [1]. Three features distinguish meiosis from mitosis: (i) a succession of two rounds of division following a single replication, (ii) pairing and recombination between homologous chromosomes, and (iii) co-segregation of sister chromatids at the first division [7] (Figure 1). In this study, we identified a gene that controls one of these three features—entry into the second meiotic division—in the sexual plant *Arabidopsis thaliana*. By combining a mutation in this gene with two other mutations—one that eliminates recombination and pairing (*Atspo11-1*) [8] and another that modifies chromatid segregation (*Atrec8*) [9]—we created a genotype in which meiosis is totally replaced by mitosis without affecting subsequent sexual processes. We called this genotype *MiMe* for “mitosis instead of meiosis”. The induction of apomeiosis by the creation of the *MiMe* genotype is an important step towards understanding and engineering apomixis.

## Results and Discussion

### *Osd1* Mutants Produce Diploid Gametes by Skipping the Second Meiotic Division

**Figure 2:**
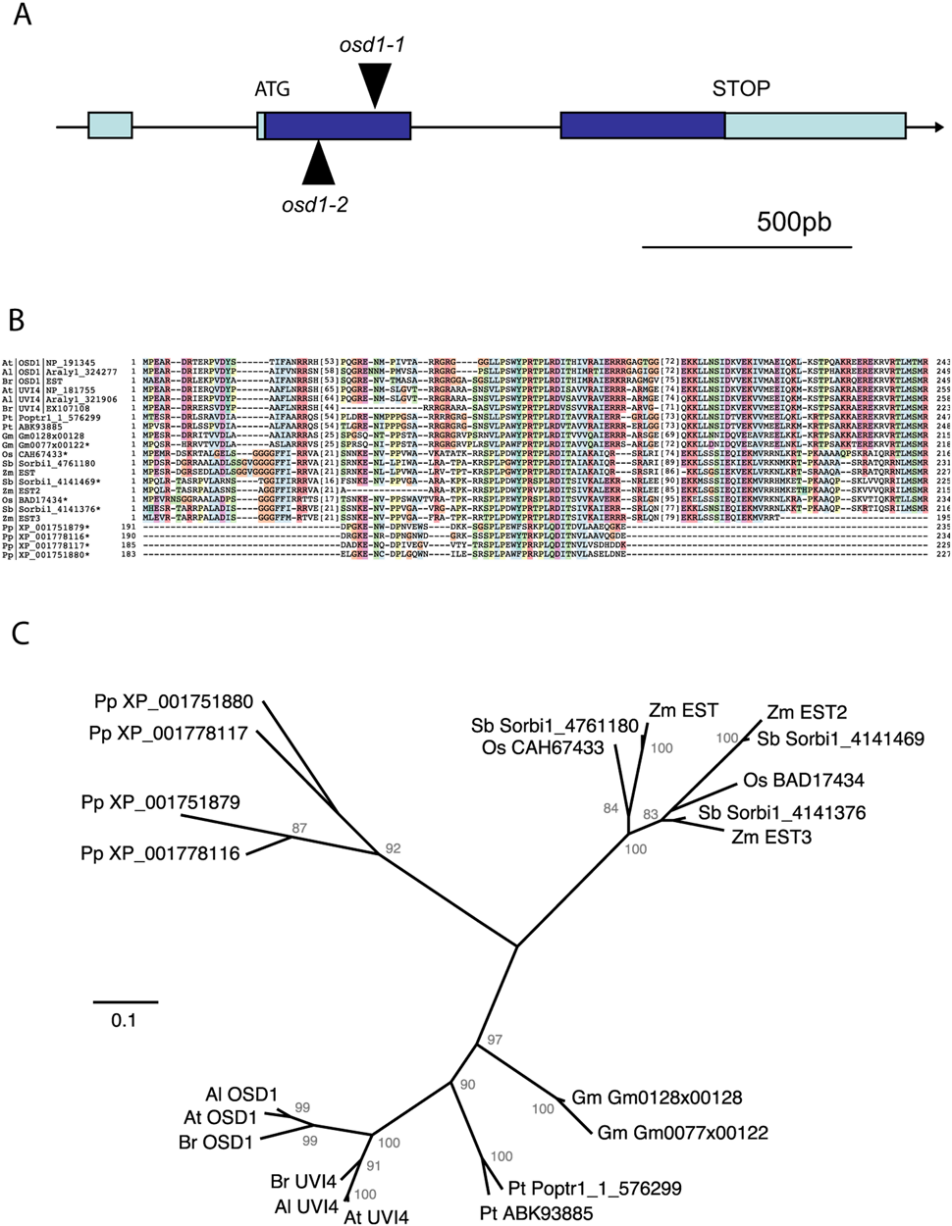
The OSD1 gene and protein. (A)The *OSD1* gene contains three exons and two introns and encodes a protein of 243 amino acids. The positions of the two Ds insertions are indicated by triangles. (B) A multiple sequence alignment of OSD1/UVI4 family members is shown, pointing out the segments of best conservation along OSD1. Particularly high conservation is observed in the central OSD1-motif, which is the only section with clear sequence similarity in moss and club-mosses. The conserved C-terminal 40–60 amino acids are predicted to contribute to alpha-helical structural elements. All available homologues are included for the selected range of species, with the species of origin being indicated by a two letter code preceding each protein identifier. Brassicaceae: Al, *Arabidopsis lyrata;* At, *Arabidopsis thaliana;* Br, *Brassica rapa.* Eurosids I: Gm, *Glycine max;* Pt, *Populus trichocarpa.* Poaceae: Os, *Oryza sativa;* Sb, *Sorghum bicolor;* Zm, *Zea mays.* Mosses: Pp, *Physcomitrella patens*. (C) Unrooted phylogenetic tree inferred from a OSD1/UVI4 family alignment using a neighbour-joining algorithm with pairwise gap removal. The full length sequences are included in the alignment except for *Physcomitrella patens* (Pp) sequences, for which only the conserved segment is aligned. The fact that only the aligning portions of Pp proteins were used in the reconstruction of the consensus tree topology should be taken into account during Pp-node branch length interpretation. Based on paralogue grouping, it can be hypothesised that gene duplication events occurred repeatedly in the evolution of plant OSD1/UVI4 family. The robustness of the topology is indicated by bootstrap confidence levels in percentage of 1,000 replicates. The analysis was performed with PHYLIPv3.66 (Felsenstein J (2006)) distributed by the author. Department of Genome Sciences, University of Washington, Seattle, United States of America) and PHYLO_WINv2.0 [32].

**Figure 3:**
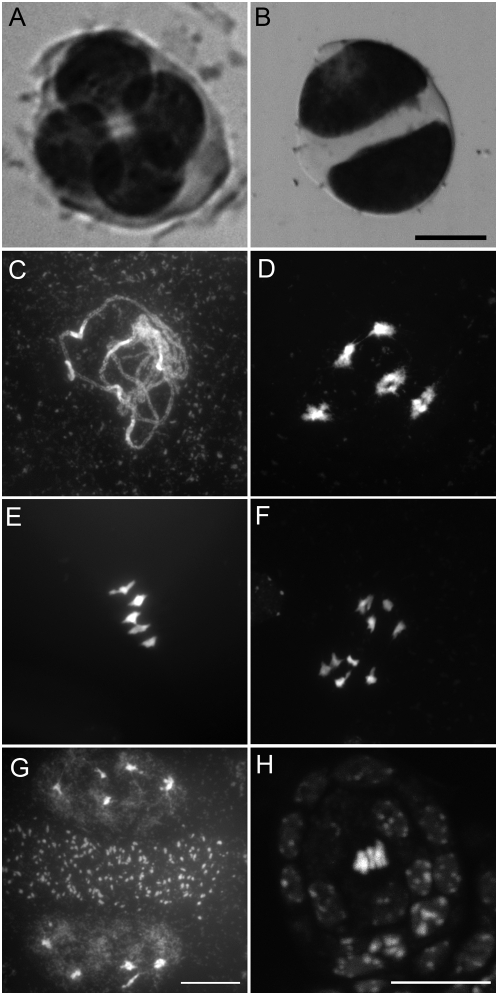
*osd1* mutants skip meiosis II. (A and B) Male meiotic products stained with toluidine blue. (A) A wild-type tetrad. (B) A dyad in the *osd1-1* mutant. (C–D) Male meiosis in *osd1* is indistinguishable from wild type until telophase I (compare to figure 4), but no figures characteristic of a second division were observed. (C) Pachytene. (D) Diakinesis. (E) Metaphase I. (F) Anaphase I. (G) Telophase I. Two nuclei separated by a band of mitochondria are observed. (H) Metaphase I of female meiosis in *osd1*.

As a part of an expression-profiling screen for meiotic genes using the Expression Angler tool [10] with the AtGenExpress tissue set [11], *At3g57860* was selected as a good candidate due to its co-regulation with several known meiotic genes [12]. *At3g57860* corresponds to the *UVI4-Like* gene (*UVI4-L*) which was briefly described in a study of its paralogue, the *UVI4* gene [13]. Due to its role in meiosis (see below), we renamed the *At3g57860* gene *OSD1,* for *omission of second division*. The OSD1 and UVI4 proteins are conserved throughout the plant kingdom (Figure 2) but do not contain any obvious conserved known functional domains. No homologues were identified outside of the plant kingdom. We investigated the role of the *OSD1* gene by isolating and characterising two mutants. The *osd1-1* (pst15307) [14] and the *osd1-2* (GT21481) [15],[16] Ds insertional mutants are in the Nooseen (No-0) and Landsberg (Ler) backgrounds, respectively, and in both cases the insertion is in the second exon of the *OSD1* gene (Figure 2). In both independent *osd1* mutants, the products of male meiosis were dyads (*osd1-1*: 714/714; *osd1-2*: 334/334) instead of tetrads (Figure 3A and 3B). Complementation tests between *osd1-1* and *osd1-2* confirmed that these mutations are allelic (*osd1-1/osd1-*2: 369 dyads/369). *osd1* mutants did not show any somatic developmental defects, male and female gametophyte lethality, or reduced fertility (wild type 38±11 seeds/fruit; *osd1* 35±6). Next, we measured ploidy levels among the offspring of diploid *osd1* mutants. Among selfed progeny, tetraploids (4n) (84%) and triploids (3n) (16%), but no diploid (2n) plants, were found (*osd1-1*: *n* = 56; *osd1-2*: *n* = 24). When mutant pollen was used to fertilise a wild-type plant, all the resulting progeny were triploid (*osd1-1*: *n* = 75). When mutant ovules were fertilised with wild-type pollen grains, we isolated 12% diploid and 88% triploid plants (*n* = 25). This demonstrated that the *osd1* mutants produce high levels of male (100%) and female (∼85%) diploid spores, which result in functional gametes.

**Figure 4:**
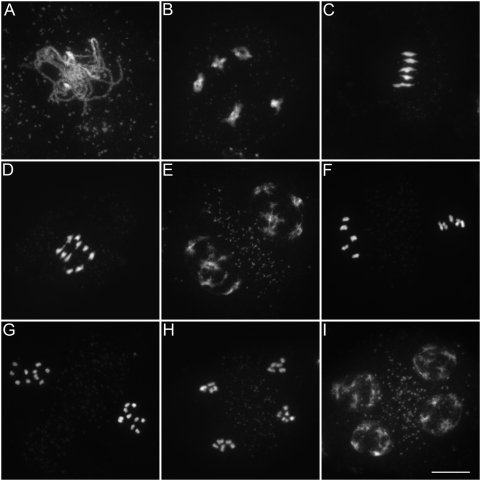
Meiosis in wild type. (A) Pachytene. Homologous chromosomes are fully synapsed. (B) Diakinesis. Five pairs of homologous chromosomes (bivalent), linked by chiasmata, are observed. (C) Metaphase I. The five bivalent are aligned on the metaphase plate. (D) Anaphase I. The homologous chromosomes are separated. (E) Telophase I. (F) Metaphase II. The pairs of sister chromatids align on the metaphase plates. (G) Anaphase II. The sister chromatids are separated. (H and I) Telophase II. Four haploid spores are formed (tetrad). Scale bar = 10 µm.

To unravel the mechanisms leading to dyad production in *osd1,* we investigated chromosome behaviour during meiosis. Both male and female meiosis I were indistinguishable from wild type (compare Figure 3 with Figure 4). Notably, chiasmata, the cytological manifestation of crossovers, and bivalents were observed. However, we were unable to find any meiosis II figures (among >500 male meiocytes from prophase to spore formation), strongly suggesting that dyad production is due to an absence of the second meiotic division. If this second division does not take place, then any heterozygosity at centromeres will be lost in the diploid gametes because of sister chromatid co-segregation and homologue separation during the first division. Because of recombination, any loci that are not linked to centromeres will segregate.

**Figure 5:**
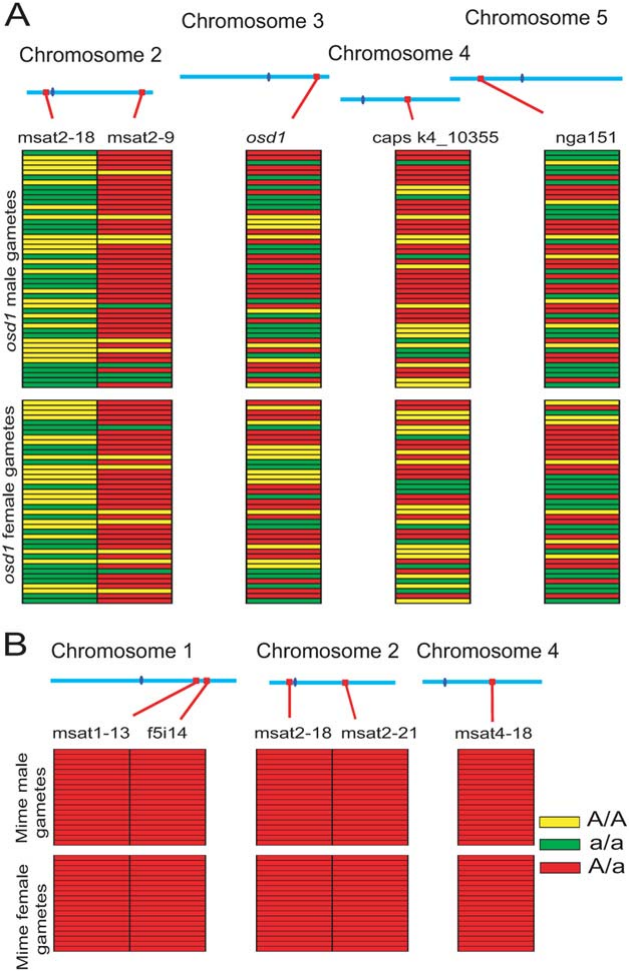
Genetic make-up of the *osd1* and *MiMe* diploid gametes. (A) Triploid offspring of the *osd-1* (No-0)/*osd1-2* (Ler) × Col-0 crosses were genotyped for several molecular markers. Each line represents one plant. For each marker, plants carrying only the No-0 allele are in green, plants carrying only the Ler allele are in yellow, and plants with both the No-0 and Ler alleles are in red. Col-0 alleles are present in all the plants because it was used as the male or female parent in the cross. The position of each marker (red) and the centromeres (dark blue) are indicated along the chromosomes. (B) Similarly, triploid offspring of the *MiMe* (patchwork of Col-0 *from Atspo11-1/Atrec8* and No-0 *from osd1-1*) × Le crosses was genotyped for molecular markers that were heterozygous in the *MiMe* mother plant. All the plants were heterozygous for each marker (Col-0/No-0).

We tested our assumption by taking advantage of the two different genetic backgrounds of the *osd1-1* (No-0) and *osd1-2* mutants (Ler). F1 plants bearing the two mutations—mutant for *osd1* and heterozygous for any No-0/Ler polymorphisms—were crossed as male or female to a third genetic background, Columbia (Col-0). Karyotyping and genotyping of the obtained plants for trimorphic molecular markers provided direct information on the genetic make-up of pollen grains and female gametophytes produced by the mutant. All the diploid gametes tested had the predicted genetic characteristics (Figure 5A). They were systematically homozygous at centromeres and segregating—because of recombination—at other loci (*n* = 48 for male diploid gametes and *n* = 41 for female diploid gametes). These results confirmed that the absence of a second meiotic division is indeed the cause of 2n gametes production in *osd1.* This mechanism also implies that unbalanced chromosome segregation at meiosis I would give rise to unbalanced dyads in *osd1*; this was confirmed by analysing a double *Atspo11-1/osd1-1* mutant (unpublished data). Such a phenotype has been already described in the maize elongate mutant in which diploid female gametes are produced because of failure to undergo meiosis II, but the corresponding gene has not been identified [17],[18].

Interestingly, the *OSD1* paralogue *UVI4* is necessary for maintaining the mitotic state, and loss of UVI4 function stimulates endo-reduplication, whereas *OSD1* is not required for this process [13]. Meiosis is not affected when *UVI4* is disrupted (unpublished data). This suggests that UVI4 and OSD1 are both involved in cell cycle regulation, with specialized functions in mitosis and meiosis, respectively. The transition from meiosis I to meiosis II requires a balance in Cyclin–Cdk activity: it must be lowered sufficiently to exit meiosis I but maintained at high enough levels to suppress DNA replication and promote entry into meiosis II [19],[20]. Two protein depletions generate a phenotype similar to that of *osd1*: the fission yeast Mes1 protein partially inhibits cyclin degradation by the anaphase promoting complex (APC) and thereby allows entry into meiosis II [21],[22]. Similarly, the expression of Erp1/Emi2 at the end of meiosis I is essential for entry into meiosis II in *Xenopus* [23],[24] and mouse [25] oocytes, most likely by inhibiting cyclin degradation by APC. Erp1/Emi2-depleted oocytes and the *mes1* yeast mutant fail to enter meiosis II, which is reminiscent of the *osd1* phenotype. Therefore, one possible function of OSD1 may also be to fine tune APC activity/cyclin levels at the end of meiosis I to ensure the transition to meiosis II.

### Turning Meiosis into Mitosis

**Figure 6:**
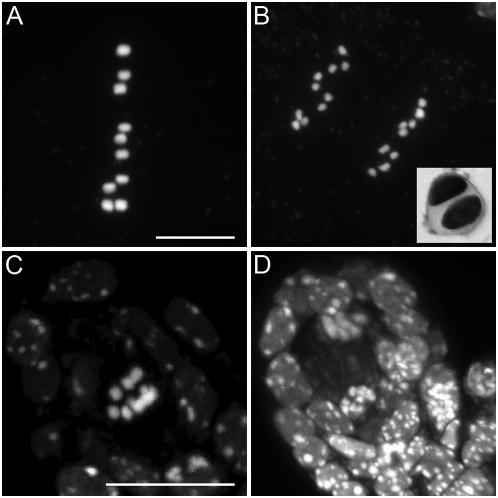
Mitosis-like divisions instead of meiosis in *MiMe* plants. (A) Male metaphase I (B) Male anaphase I. The vignette shows a dyad in *MiMe*. (C) Female metaphase I. (D) Female anaphase I. Scale bar = 10 µm.

Due to an absence of the second meiotic division, *osd1* mutants produce high frequencies of viable diploid male and female gametophytes, which generate, after fecundation, viable tetraploid plants. However, this phenomenon differs from apomeiosis in that the produced gametes are genetically different from the mother plant. Previously, we reported that in double *Atspo11-1/Atrec8* mutants, the first meiotic division is replaced by a mitotic-like division, followed by an unbalanced second division that leads to unbalanced spores and sterility [9]. Triple *osd1/Atrec8/Atspo11-1* mutants were generated and expressed an apomeiosis phenotype in which meiosis was completely replaced by a mitotic-like division. This was expected, because the *Atspo11-1* and *Atrec8* mutations lead to a mitotic-like first meiotic division, and the *osd1* mutation prevents the second meiotic division from taking place. We called this genotype *MiMe* for mitosis instead of meiosis. *MiMe* plants generate dyads (408/408) and are fertile (25±6 seeds per fruit). The *osd1* mutation therefore suppressed the sterility phenotype of the *Atspo11-1/Atrec8* double mutant. Observation of chromosome behaviour during male and female meiosis revealed a mitotic-like division: ten univalents aligned on the metaphase plate and sister chromatids separated at anaphase (Figure 6). The selfed progeny of *MiMe* plants were systematically tetraploid (*n* = 24), and backcrosses between diploid *MiMe* plants and wild-type plants generated triploid plants regardless of whether male (*n* = 24) or female (*n* = 67) *MiMe* gametes were used, showing that this mitotic-like division gives rise to functional diploid gametes. All the gametes (male and female), tested similarly as described above systematically retained the mother plant heterozygosity for every genetic marker tested (Figure 5B) and were thus genetically identical to the mother plant. These results confirm that *MiMe* plants undergo a mitotic-like division instead of a normal meiotic division, without affecting subsequent sexual processes.

**Figure 7:**
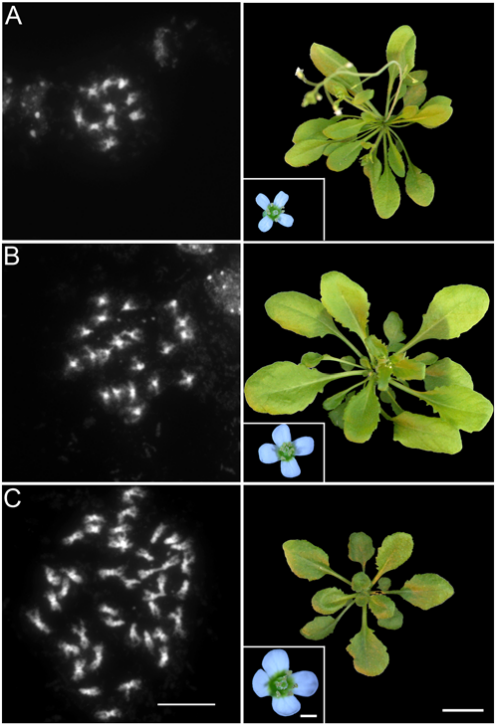
Doubling of ploidy at each generation in the *MiMe* line. In subsequent generations, *MiMe* plant ploidy doubled at each generation, from 2n (A) (10 chromosomes), to 4n (B) (20 chromosomes) and 8n (C) (40 chromosomes). The left column shows mitotic metaphase, scale bar = 10 µm. The right column shows the corresponding four-week-old plants, (scale bar = 2 cm) and flowers (scale bar = 1 mm).

**Figure 8:**
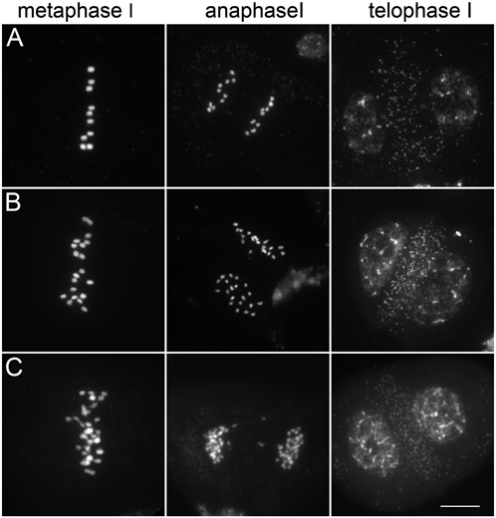
Male meiosis in 2n, 4n, and 8n *MiMe* plants. *MiMe* plants underwent a mitosis-like division instead of meiosis in 2n (A), 4n (B), and 8n (C) plants. Meiotic metaphase I, anaphase I, and telophase I are shown for each generation. Scale bar = 10 µm.

When meiosis is replaced by mitosis, ploidy is expected to double with each generation. Indeed, in successive generations, we obtained tetraploid (4n, 20 chromosomes, *n* = 26) and octoploid (8n, 40 chromosomes, *n* = 33) (Figures 7 and 8). Fertility dropped from 25±6 seeds/fruit in 2n plants and 19±4 in 4n plants to <0.1 in 8n plants. Further investigations will be required to understand the cause of this reduced fertility associated with high ploidy level.

### Towards Apomixis

Apomixis can be separated into three developmental components: an absence or alteration of meiosis which prevents reduction (apomeiosis), the fertilization-independent development of the embryo from the egg cell (parthenogenesis), and the initiation of endosperm development with or without fertilization [1]. Here we showed that fully penetrant apomeiosis can be induced in a sexual plant, when a mitotic-like division replaces meiosis in the *MiMe* genotype. A previous case of apomeiosis was reported in the *Arabidopsis dyad* mutant [26]. However, only 0.2% of *dyad* ovules generate viable gametes, which makes it practically unusable in an apomixis engineering strategy, in contrast to *MiMe* plants, which produce quasi–wild type levels of viable—and apomeiotic—ovules and pollen grains. The three genes conferring the *MiMe* genotype are strongly conserved among plants, suggesting that apomeiosis may be engineered in any plant species, including crops. In addition, several other genes required for recombination initiation have been described in plants [27], and the corresponding mutants could be used instead of *spo11-1* to construct a *MiMe* genotype. However, it remains to be confirmed that the *MiMe* genotype would have the same phenotype in other plant species. To obtain apomixis, in addition to apomeiosis, parthenogenesis will have to be introduced, and the problem of endosperm formation must also be overcome. However, mutations that mimic early parthenogenesis or give rise to functional autonomous endosperm have been reported in *Arabidopsis* [28],[29], suggesting that it should be ultimately feasible to introduce apomixis into a sexual plant species.

## Materials and Methods

### Growth Conditions

*Arabidopsis* plants were cultivated as described in [30]. For germination assays and cytometry experiments, *Arabidopsis* were cultivated in vitro on *Arabidopsis* medium [31] at 21°C with a 16-h day/8-h night photoperiod and 70% hygrometry.

### Genetic Analysis

Plants were genotyped by PCR (30 cycles of 30 s at 94°C, 30 s at 56°C, and 1 min at 72°C) using two primer pairs (see Table 1). For each genotype, the first primer pair is specific to the wild-type allele and the second pair is specific to the insertion.

**Table 1:** Primers for mutant genotyping.

*mutant*	Primers for Wild-type Allele	Primers for Mutant Allele
*osd1-1*	pst15307U (CGTCACTCTCCCCAAGAAAG3)	pst15307L
	pst15307L (GGCTAAGCAAGCCTGCTATG3)	Ds5-2a (TCCGTTCCGTTTTCGTTTTTTAC).
*osd1-2*	GT21481U (CCGGTGTTCTTGTGACTCG)	GT21481U
	GT21481L (GCAGATTCCTAATTCAGCTC)	Ds3-4 (CCGTCCCGCAAGTTAAATATG)
*Atspo11-1-3*	N646172U ( AATCGGTGAGTCAGGTTTCAG )	N646172L
	N646172L (CCATGGATGAAAGCGATTTAG)	LbSalk2 (GCTTTCTTCCCTTCCTTTCTC)
*Atrec8-3*	N836037U (CTCATATTCACGGTGCTCCC)	N836137L
	N836037L (GGGGGAAAAGAGAAAGGTTC)	LB3sail (TAGCATCTGAATTTCATAACCAATCTCGATACAC)

Genetic trimophic (Col-0/Ler/No-0) markers used to genotype the *osd1-1* (No-0)*/osd1-2* (Ler) × Col-0 F1 population and *osd1-1* (No-0)*/spo11-1* (Col-0)/*rec8* (Col-0) triple mutant × Ler F1 population were amplified (40 cycles of 30 s at 94°C, 30 s at 58°C, and 30 s at 72°C) with specific primers (see Table 2) and observed after migration on 3% agarose gel. CAPS K4_10355 was observed after Eco47III/HpaII double digestion. The two primer pairs specific for the *osd1-1* and *osd1-2* insertion borders were used as a marker on Chromosome 3.

**Table 2:** Genetic markers.

Marker	Chromosome	Position (bp)	Primer 1	Primer 2
Msat1-13	1	25,827,433	CAACCACCAGGCTC	GTCAAACCAGTTCAATCA
F5i14	1	24,374,008	CTGCCTGAAATTGTCGAAAC	GGCATCACAGTTCTGATTCC
Msat2-18	2	2,799,644	TAGTCTCTTTTGGTGCGCATA	AGCCTCTCCAAGCTTAGGTCT
Msat2-21	2	11,461,020	ATTTTTAGCCCAATCACGTTT	AGGTCAAGTGAAAGGGTAAGG
Msat2-9	2	18,152,580	TAAAAGAGTCCCTCGTAAAG	GTTGTTGTTGTGGCATT
CapsK4_10355	4	10,354,800	ACCCATTTGGTGATGCTAAC	GAGCAGTTTCCACTTTGTCC
Msat4-18	4	11,966,304	TGTAAATATCGGCTTCTAAG	CTGAAACAAATCGCATTA
Nga151	5	4,669,932	GTTTTGGGAAGTTTTGCTGG	CAGTCTAAAAGCGAGAGTATGATG

### Cytology and Flow Cytometry

Final meiotic products observation, chromosomes spreads, and genome size measurement were carried out using the techniques described by d'Erfurth et al. [12].

## Abbreviations

APC: anaphase promoting complex
Col-0: Columbia background
Ler: Landsberg background
MiMe: mitosis instead of meiosis
No-0: Nooseen background
